# Pneumoscrotum: A Rare Presentation of Gastric Perforation in a Neonate

**Published:** 2010-12-01

**Authors:** Yousuf Aziz Khan, Jamshed Akhtar

**Affiliations:** Department of Pediatric Surgery, National Institute of Child Health Karachi, Pakistan

**Keywords:** Gastric perforation, Pneumoscrotum, Neonate

## Abstract

Pneumoperitoneum in neonates is not an uncommon condition. Free air in peritoneum may be secondary to host of pathological lesions. Usually the patient presents with signs of intraperitoneal sepsis, however presence of air in the scrotum as a concomitant sign is a rare event. Herein we report a 4-day-old neonate who presented with 2 days history of fever and scrotal swelling. Abdominal signs were subtle. Scrotum was hugely distended and tense. Workup of the patient revealed free intraperitoneal gas with air in the scrotum. At exploration, two perforations were found near the greater curvature of stomach and repaired. Post-operative course was uneventful.

## INTRODUCTION

Free intraperitoneal gas on abdominal x-rays, the pneumoperitoneum, is not an uncommon presentation in neonates. Mostly it is taken as a sign of gut perforation and laparotomy is generally required [1]. It may also be found in neonates with severe respiratory distress, after aggressive resuscitation or with mechanical ventilation [1-4].

Pneumoperitoneum presenting as pneumoscrotum secondary to gastric perforation without abdominal findings is rare. We report a case of neonate who presented with scrotal swelling and was found to have gastric perforations at laparotomy. 

## CASE REPORT

Four days old (1.8 kg) full term male neonate presented with fever and gross scrotal swelling for two days. The baby was delivered at home and nothing significant found in perinatal history. He passed meconium within few hours of birth and had no vomiting.

On examination, the baby was febrile (100 ºF), irritable, reluctant to feed, with heart rate of 130/min, and respiratory rate of 42/min. Chest was clear on auscultation and abdomen was non distended and soft. The umbilical stump was infected. There was a huge, irreducible, soft swelling at the right hemi-scrotum, extending proximally to the inguinal region. The scrotal skin was tense and shiny (Fig. 1). Right testis could not be palpated. Initially a diagnosis of irreducible inguinal hernia was made.

**Figure:**
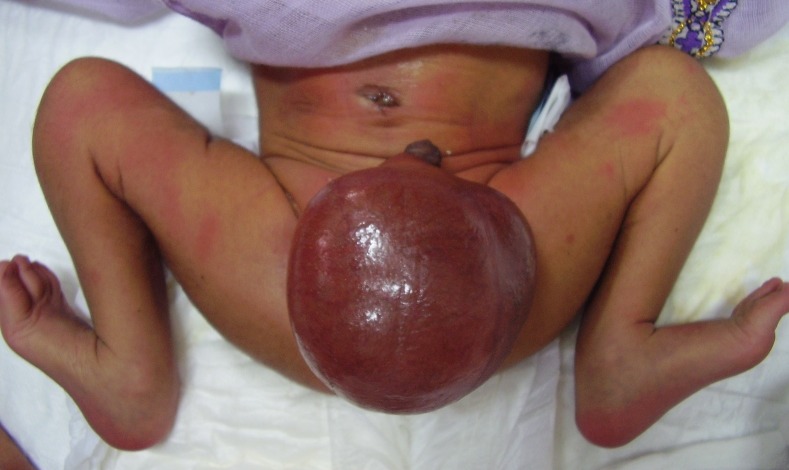
Figure 1: Newborn with grossly distended scrotum with thin shiny skin (scrotal pneumatocele) with normal looking abdomen.

Laboratory parameters were within normal limits. X-ray abdomen and pelvis in erect posture revealed free gas under the right dome of diaphragm and in the scrotum (Fig. 2). With the suspicion of gut perforation, laparotomy was performed.

**Figure:**
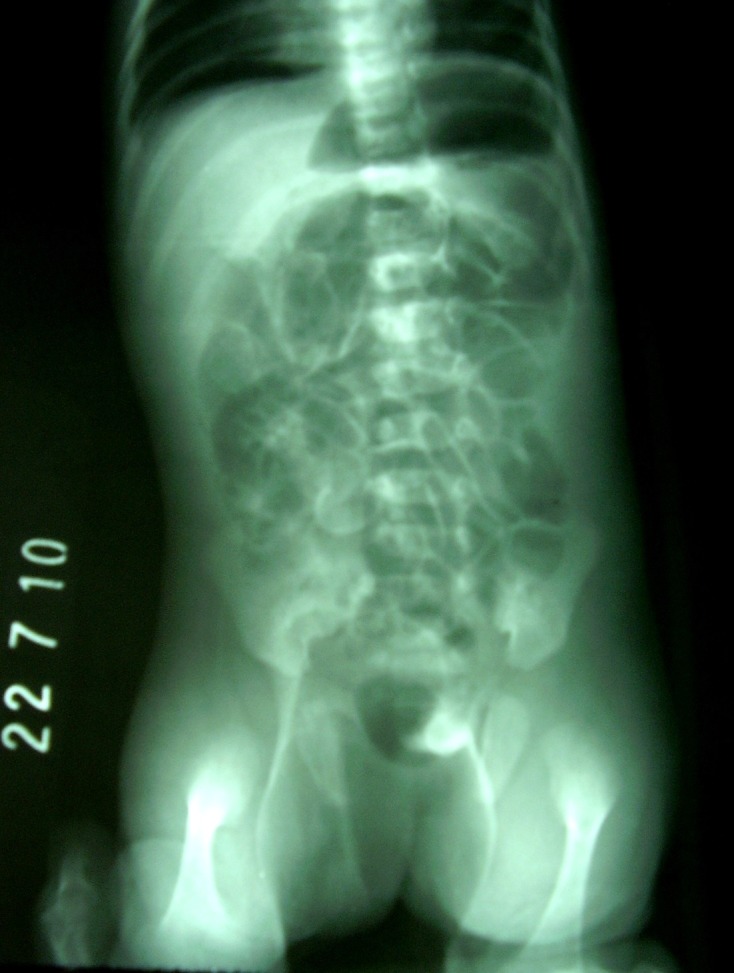
Figure 2: X-ray Abdomen (erect) showing free gas in the peritoneum (under the right dome of diaphragm) and free gas in the scrotum.

The scrotal swelling spontaneously reduced as the peritoneal cavity was opened. Two small perforations were found at the posterior wall of stomach near the greater curvature. Rest of the peritoneal cavity was clean and gut appeared healthy. Primary repair of the perforations was done. Post-operative course was uneventful. Baby was allowed orally on 6th post-operative day and discharged on 8th day.

## DISCUSSION

Pneumoperitoneum in neonates has multiple causes and varied presentations ranging from a vitally stable newborn with minimal or no abdominal signs to a sick baby with gross abdominal distension. Mostly it is taken as a sign of gut perforation. Rarely, extra abdominal cause may be the source of free intraperitoneal air [2-4]. Very rarely it may be without any surgical or medical cause, termed as benign pneumoperitoneum [1].

Gastric perforation in neonates is a rare event and represents immediate surgical emergency. It usually occurs in premature neonates in intensive care setting. Proposed mechanisms of this perforation are trauma, ischemia, and one occurring spontaneously [5]. Traumatic perforations are usually iatrogenic, occurring due to vigorous nasogastric tube placement or massive gastric distension associated with aggressive resuscitation with bag mask ventilation or in newborns with respiratory distress and on mechanical ventilation [5]. Severe birth asphyxia, prematurity and sepsis are the common risk factors associated with ischemic perforations of stomach in newborns [6]. Spontaneous perforations occur usually in healthy newborns without gastrointestinal conditions, presenting within 2 - 7 days of life. No exact predisposing or risk factor can be identified in such newborns. Rarely congenital muscular deficiency of stomach may be the cause of gastric perforation. It has also been reported secondary to distal obstruction and atresias [6]. No such etiological factors were found in our patient and it may be suggested that the gastric perforations were spontaneous in nature.

Clinically, a newborn with gastric perforation presents with gross abdominal distension compromising ventilation along with signs of hypovolemic shock and sepsis and radiologically, massive pneumoperitoneum is noticed [5, 6]. In our case, the baby had clinically normal abdomen and very small amount of free intraperitoneal air on x-ray.

Although pneumoscrotum has been reported secondary to perforation of Meckel’s diverticulum, perforation of ileum secondary to atresia, after aggressive resuscitation, and with mechanical ventilation; scrotal pneumatocele secondary to gastric perforations and without signs of intraperitoneal sepsis is rarely reported. As processus vaginalis is patent in 80 - 95 % newborn males, free air in the peritoneum passing through the patent process vaginalis, causing gaseous distension of the scrotum is the possible explanation of pneumoscrotum in this case. Other mechanisms postulated in the development of pneumoscrotum are subcutaneous or retroperitoneal air dissecting down the dartos lining of the scrotal cord into the scrotal wall or may result from local production of gas secondary to infections [3, 6-10].

In conclusion, pneumoscrotum along with pneumoperitoneum without significant abdominal distension may be a presentation of neonatal gastric perforation and demands high index of suspicion to manage the condition effectively.

## Footnotes

**Source of Support:** Nil

**Conflict of Interest:** None declared
